# Progressive Adaptation in Physical Activity and Neuromuscular Performance during 520d Confinement

**DOI:** 10.1371/journal.pone.0060090

**Published:** 2013-03-28

**Authors:** Daniel L. Belavý, Ulf Gast, Martin Daumer, Elena Fomina, Rainer Rawer, Hans Schießl, Stefan Schneider, Harald Schubert, Cristina Soaz, Dieter Felsenberg

**Affiliations:** 1 Centre of Muscle and Bone Research, Charité Universitätsmedizin Berlin, Berlin, Germany; 2 Sylvia Lawry Centre for Multiple Sclerosis Research e.V. - The Human Motion Institute, Munich, Germany; 3 Institute for Biomedical Problems, Russian Academy of Sciences, Moscow, Russia; 4 Novotec Medical Gesellschaft mit beschränkter Haftung, Pforzheim, Germany; 5 Institute of Movement and Neurosciences, German Sport University, Cologne, Germany; 6 Institute for Data Processing, Technische Universität München, Munich, Germany; University of Las Palmas de Gran Canaria, Spain

## Abstract

To understand whether prolonged confinement results in reductions in physical activity and adaptation in the musculoskeletal system, six subjects were measured during 520 d isolation in the Mars500 study. We tested the hypothesis that physical activity reduces in prolonged confinement and that this would be associated with decrements of neuromuscular performance. Physical activity, as measured by average acceleration of the body’s center of mass (“activity temperature”) using the actibelt® device, decreased progressively over the course of isolation (*p*<0.00001). Concurrently, countermovement jump power and single-leg hop force decreased during isolation (*p*<0.001) whilst grip force did not change (*p*≥0.14). Similar to other models of inactivity, greater decrements of neuromuscular performance occurred in the lower-limb than in the upper-limb. Subject motivational state increased non-significantly (*p* = 0.20) during isolation, suggesting reductions in lower-limb neuromuscular performance were unrelated to motivation. Overall, we conclude that prolonged confinement is a form of physical inactivity and is associated with adaptation in the neuromuscular system.

## Introduction

The effect of prolonged isolation, or confinement, on the musculoskeletal system has previously not been studied. Confinement studies have to date examined psychosocial [1], biomedical [2], skilled motor [2], [3] and immune system [4] variables. Aside from the most common case of prolonged confinement, imprisonment, individuals are also confined as part of over-wintering in isolated regions of the Earth [5], military operations [3], [6] and ground based spaceflight operation simulation [1].

It is conceivable that confinement will result in a reduction of physical activity, though this idea has not yet been formally tested. We know from studies of physical inactivity and immobilization, such as after bone fracture [7], stroke [8], spinal cord injury [9] and prolonged bed-rest [10], that the reduction of loading results in regional decrements of peak neuromuscular performance, regional losses of bone density and muscle mass. We therefore hypothesized that prolonged confinement in otherwise healthy individuals would result in a reduction in physical activity and consequently a loss of neuromuscular function in the lower-limbs. To evaluate neuromuscular function we prefer to measure complicated tasks, such as countermovement jumping and single-leg hopping rather than, for example, isolated maximal force production at individual joints. For example, with age [11] greater declines of countermovement jump performance are seen than of isometric knee extension. The ability of an individual to jump and hop requires them to integrate a wider variety of systems, such as the ability to store energy in passive structures, generate force via the musculature, control body and limb posture.

The Mars500 project was implemented to simulate a flight to Mars. In this project, participants were confined for 520 days with only video and email communication with the outside world. This project provided the opportunity to test our hypotheses.

## Materials and Methods

### Mars500 Study

Six healthy male subjects (three Russian, two European, one Chinese; 32.4(4.8)years, 176(4)cm and 84.9(8.9)kg) participated in the Mars500 study (http://mars500.imbp.ru/en/index_e.html). Subjects attended the Institute of Biomedical Problems in Moscow, Russia from May 2009 to November 2011. After an initial four week baseline data collection period, subjects entered the isolation capsule for a period of 520 days on the 3^rd^ June 2010. Subjects remained at the facility for two weeks for post-isolation testing. Subjects did not leave the isolation capsule for the entire 520 days and all supplies (i.e. food and other consumables) were placed inside the capsule prior to the start of isolation. Protocols were set in place in case of medical and other emergencies, but did not need to be implemented. This research was conducted in accordance with the principles expressed in the Declaration of Helsinki. The ethical committee of the Institute of Biomedical Problems approved this study and subjects gave their written informed consent.

The aim of the Mars500 study was to simulate the isolation during a flight to Mars. Communication was possible via email or video message only and a time-delay in communication of up to 20 minutes was introduced according to the simulated distance from “Earth”.

For each measurement protocol, one subject was selected to be responsible for its implementation during the project. Before isolation, he was taught how to implement the tests, was responsible for coordinating testing of the remaining subjects during isolation and was able to test himself. Data collected during isolation was copied onto USB-stick and was transferred to the outer world via the rubbish disposal unit.

### Physical Activity Monitoring via the Actibelt® System

**Figure 1:**
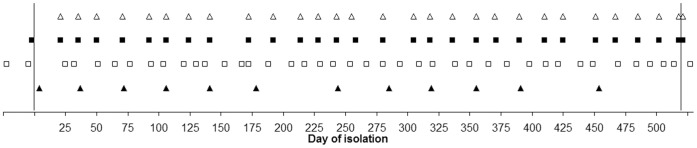
Testing schedule. Δ: maximal grip force testing. ▪: countermovement jump and multiple single-leg hop tests; ▴: first of five days wearing the actibelt**®** device for activity level measurements. □; questionnaires for assessment of motivational state. Vertical lines indicate the start and end of the isolation phase.

Subject physical activity was monitored using the actibelt® (The Human Motion Institute, Munich, Germany; [12], [13]) thirteen times during isolation with the first measurement session beginning on the fourth day of isolation (Figure 1). At each measurement time-point, subjects wore the actibelt®, a three dimensional accelerometer with 100 Hz sampling rate, on their belt for six consecutive days. The device was also worn at night-time. The variables of “activity temperature”, distance ambulated and average gait speed were computed:

#### Activity temperature

Measured the average absolute acceleration of the body’s center of mass, with the effect of gravity removed, over a period of time. It was calculated as the average of the “activity counts” over the recording time (Unit: *g* [9.81 m/s^2^]). This variable was calculated according to the formula:
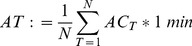
(1)Where *N* was the number of minutes in the time-period to be analysed (i.e. 1440 in a 24-hour period). AC was the “activity count” per minute. To calculate AC, the three acceleration axes 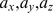 were combined and the effect of gravity was removed according to the formula:
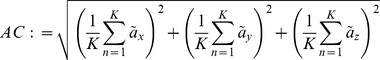
(2)Where K was the number of data points per minute and: 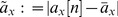
(3)

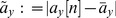
(4)

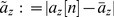
(5)Where 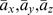 was an approximation of the mean calculated by a low pass first order Butterworth filter with cut-off frequency of 1/6 Hz.

#### Distance ambulated

Total distance travelled, calculated as the sum of all step lengths (unit: m). Length of each step was calculated as described in a previous publication [13].

#### Average gait speed

Gait speed per step was calculated as described previously [13]. Average gait speed was then calculated as the average speed across all steps taken (unit: m/s).

The variables were calculated for each continuous 24-hour period of data collection. 24-hour periods of data collection ran from midnight of the previous day to midnight of the current day minus one millisecond. Data from the four days of continuous data collection, days one to five of each measurement session, were averaged prior to further analysis.

### Countermovement Jump, Single Leg Hopping and Grip Force Testing

#### Countermovement jump

The first measurement was performed prior to isolation, with twenty-eight testing sessions during isolation and one final test three days after isolation (Figure 1). Maximal countermovement jump testing was performed on a ground reaction force platform (Leonardo Mechanograph GRFP, Novotec GmbH, Pforzheim, Germany). Subjects first stood on the 66×66 cm testing platform with their arms resting at their sides. Before the first jump, the subject’s body mass was measured for use in subsequent calculations. Subjects were then instructed to perform a countermovement (i.e. a brief squat beforehand) jump and jump as high as possible. Three jumps were performed during each testing session with a break of 1 minute between each jump. Software provided by the manufacturer (Leonardo Mechanography v4.2) was used for recording and storage of data and for subsequent calculation of the variables of interest. The peak power and maximum jump height were calculated. The data from the jump of maximal jump height from all three trials was used in further analysis.

#### Multiple single-leg hopping

The same testing apparatus was used as for the countermovement jump and test were performed at the same time-points of the study. This test aimed to examine the force development at the ankle joint [14]. In the course of ten continuous single leg hops, the subject was required to maintain their knee close to full extension and no arm swing was permitted. Subjects began the test in relaxed standing, shifted their weight onto one leg, and the subject was instructed to perform begin the ten consecutive maximal hops. Subjects were instructed to maintain the heel off the ground for all hops. Each leg was tested twice with a break of 1 minute between test runs. Using the same software as per the countermovement jump test, hop force and hop force per unit body weight were calculated. Data from the single hop of the highest force from the two sets of ten consecutive hops from both legs was chosen for further analysis.

#### Maximal grip force

Testing was performed in standing using a digital hand dynamometer (Takei Scientific Instruments Co. Ltd, Tokyo, Japan). The first measurement was done on the 21^st^ day of isolation with twenty-eight subsequent measurements (Figure 1). The shoulder was placed in an adducted and neutrally rotated position with the elbow in full extension [15]. Three repetitions were performed on both hands with a 30 second break between tests. To increase measurement precision, the average value (in Newtons) of the three measurements was used in further analysis.

### Motivational State

To assess whether any reductions in subject motivation may occur during isolation and whether this may relate to changes in other variables, motivational state was assessed. This methodology has been described in detail elsewhere [16]. However, in brief, the subject was presented with eight adjectives. Each subject completed a questionnaire in their native language (Russian, Chinese, Italian or French). The subject was required to indicate whether a given adjective described their physical or mental state on a six level ordinal scale from 0 (not at all) to 5 (totally). The scores assigned by the subjects to these adjectives built the motivation sub-dimensions of ‘willingness to seek contact’ (open for contact, communicative), ‘social acceptance’ (accepted, popular), ‘readiness to strain’ (energetic, powerful) and ‘self-confidence’ (experienced, self-confident). The score for ‘motivational state’ was then calculated as the average score across these four sub-dimensions. This test was performed twice before isolation, thirty-eight times during isolation and once after isolation (Figure 1). Data from the two tests before isolation were averaged prior to further analysis.

### Statistical Analysis

Linear mixed-effects models [17] were used to assess each parameter. A main-effect of study-date was included in the models. Allowances for heterogeneity of variance due to study-date were made where necessary. Analysis of variance (ANOVA) was then performed for each model. Subsequently a priori comparisons were conducted comparing the changes over time to baseline measurements.

For the variable of motivational state a non-parametric analog of repeated measures ANOVA was performed to assess the changes over time [18]. The ANOVA-type test statistic is reported for these data. Wilcoxon-tests were also performed comparing the data on each day during and after isolation to the baseline data.

An alpha level of 0.05 was taken for statistical significance. An adjustment for multiple comparisons was not performed. The “R” statistical environment (version 2.10.1, www.r-project.org) was used for all analyses. Unless otherwise stated, all values are reported as mean(SD).

Based upon the greatest changes seen during isolation for the actibelt® activity temperature (effect size: −2.73) and countermovement jump peak power (effect size: −1.42) a post-hoc power analysis (simple “difference from zero” one sample t-test, two-tailed, n = 6, alpha-level: 0.05; computed with G*Power3; [19]) showed an achieved power of, respectively, 0.9993 and 0.79.

## Results

The baseline data are given in Table 1. All subjects completed the isolation phase.

**Table 1:** Physical activity and neuromuscular variables at first measurement.

Test	Parameter	Value
*Physical activity: actibelt®*	Average gait speed	1.12(0.16) m/s
	Distance ambulated	3686(1679) m
	Activity temperature	4.64(0.68) g
*Countermovement jump*	Power	4.07(0.51) kW
	Power relative to body mass	48.0(5.8) W/kg
	Height	0.43(0.07) cm
*Single-leg hopping*	Force	2.66(0.23) kN
	Force relative to body mass	3.22(0.32) g
*Grip force*	Left hand	46.1(6.0) N
	Right hand	47.7(6.0) N

Values are mean(SD). The first test of physical activity, countermovement jump and single-leg hopping were conducted before isolation. Grip force was first measured on the 21^st^ day of isolation. *g* = 9.81 m/s^2^. Activity temperature is a measure of the average absolute acceleration over the course of the day: more changes in body position (e.g. sit-stand) and more changes in walking speed means higher overall average acceleration.

### Physical Activity Levels

**Figure 2:**
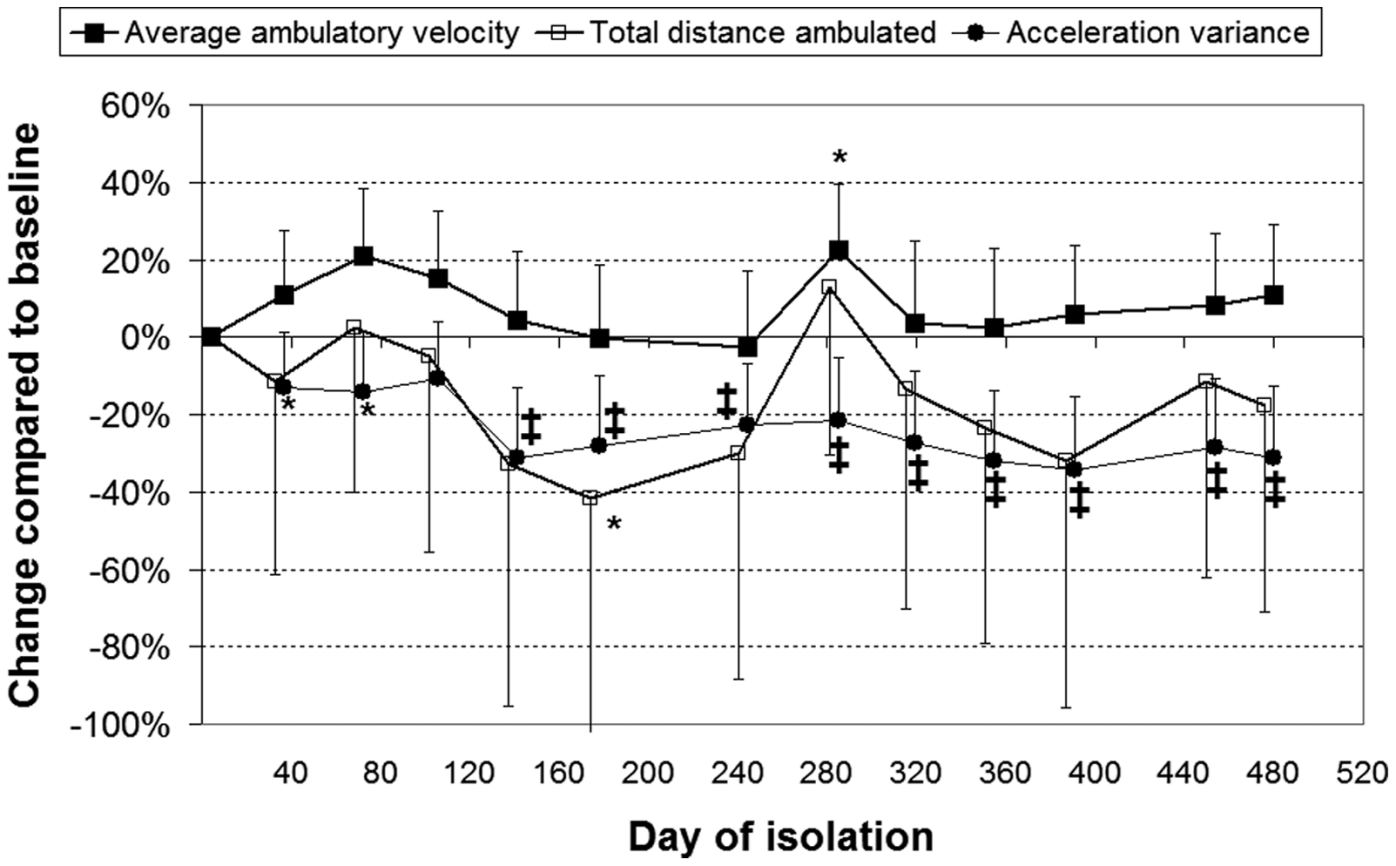
Physical activity during isolation. Values are mean (SD) percentage change compared to first measurement. *: *p*<0.05; †: *p*<0.01; ‡: *p*<0.001 and give the significance of the change. The variables measured at the same time-points have been offset slightly. Average gait speed and distance walked were significantly increased when subjects were scheduled to perform running exercises (days 71–141, 285–354 and 451–520 of isolation; statistical analyses not shown).

All measurement sessions during isolation were completed with the exception of incomplete data in one subject from the third measurement time-point. Significant changes occurred in the activity temperature (*p*<0.00001) whereas the changes in distance ambulated (*p* = 0.07) and average gait speed (*p* = 0.08) did not reach significance on ANOVA. After the first measurement beginning on the fourth day of isolation, activity temperature (variation of acceleration of the body’s center of mass) reduced (Figure 2). The distance walked decreased marginally and this effect only reached significance at day-180 of isolation. At around day 280 of isolation, a sudden increase in ambulatory velocity and distance ambulated was seen compared to the prior and following testing sessions. This testing session was immediately after the simulated “departure” from Mars during the isolation study.

### Countermovement Jump, Single Leg Hopping and Grip Force Testing

**Figure 3:**
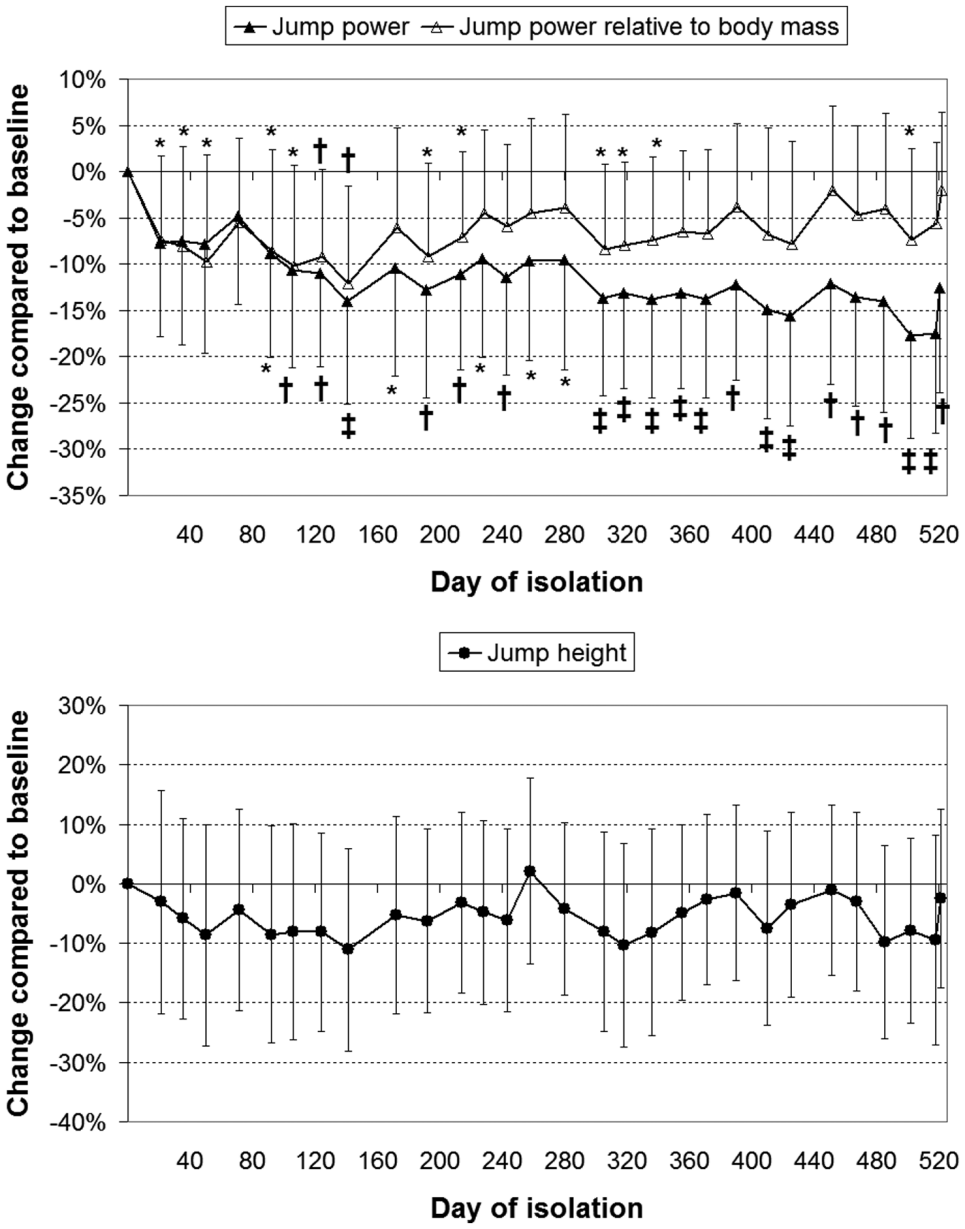
Reductions in countermovement jump power (top) but not height (bottom). Values are mean (SD) percentage change compared to first measurement. *: *p*<0.05; †: *p*<0.01; ‡: *p*<0.001 and give the significance of the change.

**Figure 4:**
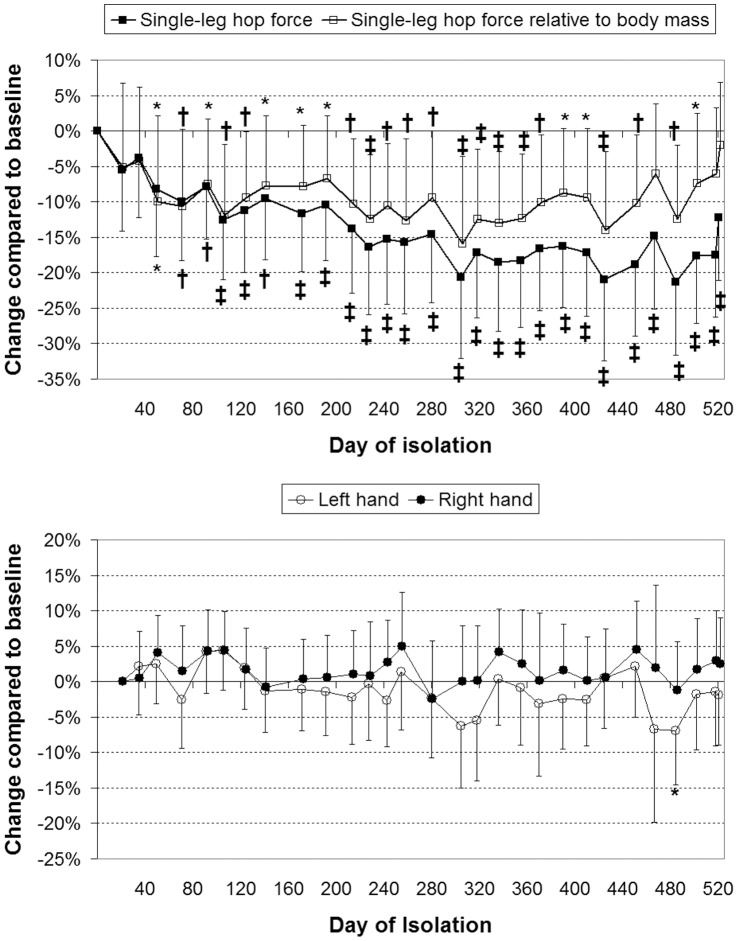
Reductions in single-leg hop force (top) but not grip force (bottom). Values are mean (SD) percentage change compared to first measurement. *: *p*<0.05; †: *p*<0.01; ‡: *p*<0.001 and give the significance of the change.

Countermovement jump power and power relative to body mass both reduced significantly (*p*<0.001) over the course of the study. The reductions in jump power relative to body mass were lesser in magnitude (Figure 3). Although ANOVA indicated a significant change in maximum jump height over the course of the study (*p* = 0.005), inspection of the data indicated that, whilst marginally reduced, no significant changes occurred in peak jump height compared to baseline (Figure 3). Single-leg hop force, and force per unit body mass, both reduced (*p*<0.001) over the course of the study (Figure 4). ANOVA indicated that grip force did not change significantly during isolation (*p*≥0.14; Figure 3).

### Motivational State

**Figure 5:**
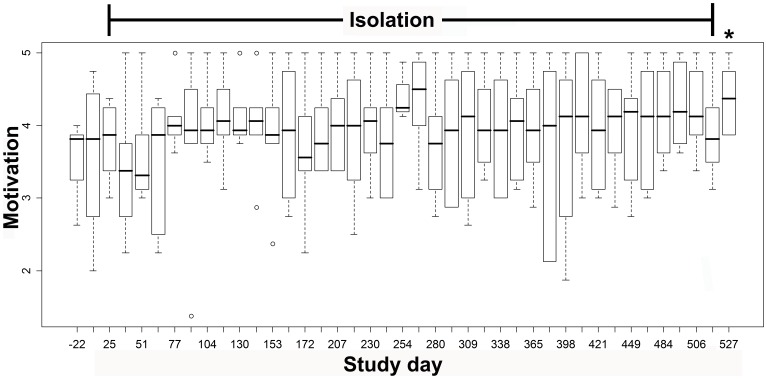
Boxplots of subjective motivation level over the course of isolation. Non-parametric form of repeated measures analysis of variance showed no significant changes over time (*p* = 0.20). *: *p* = 0.045 on Wilcoxon-test versus pre-isolation.

Subject motivation levels tended to increase during isolation (Figure 5) but this effect was not significant on non-parametric ANOVA (*p* = 0.20).

## Discussion

The current study investigated male subjects involved in 520 d confinement. To the best of our knowledge no prior work has quantified physical activity and neuromuscular performance in this model. We found that physical activity, when measured in terms of average acceleration, reduced. This indicates that subjects changed body position less and/or changed their speed of walking less. Although the effects did not reach statistical significance, distance ambulated decreased marginally and average gait speed stayed the same or increased marginally. Explosive power during countermovement jump and force production during single-leg hopping, a test influenced predominately by the ankle joint plantarflexors [14], also reduced progressively during confinement and these effects were still present after controlling for changes in body mass. Grip force showed little change.

The findings from the actibelt® measure show that a reduction of physical activity occurred during confinement. Whilst the average gait speed remained the same and the distance ambulated decreased marginally, there was a strong significant reduction in the acceleration of the body’s center of mass. This implies that there was a reduction in the forces generated within the body – be it from changes in body position or other forms of acceleration.

In the assessment of neuromuscular function in the lower- and upper-limbs, we observed a pattern of greater decrements in neuromuscular performance in the lower-limb (countermovement jump power, multiple single-leg hopping) with little change in the upper-limb (grip force). It is possible that these changes occurred due to the new “lifestyle” of reduced physical activity in confinement. In models of extreme disuse, such as bed-rest and spaceflight, it is well known that greater loss in function, muscle mass and bone density occur in the lower-limbs with limited changes in the upper-limbs [20]. With age, decline in peak countermovement jump power per unit body weight occurs in both elite athletes [21] and the wider population [22], though the rates of decline seen in these earlier works were slower than seen in the current study. Associated with such decline, a number of mechanisms including inhibition of muscle regeneration via satellite cells [23], [24] and oxidative stress [25], [26] have been identified.

There are, however, some aspects of the finding of the current study do not support the interpretation that the changes in neuromuscular function are due to reduced physical activity. For example, the main reductions in countermovement jump power and single-leg hop force relative to body mass occur at around 40–80 days of confinement, with some indication of progressive losses beyond this time-point. This aspect of the findings does not support the idea of a 1∶1 relationship between changes in activity levels and adaptation in lower-limb neuromuscular function.

The effects we observed during isolation were likely not due to changes in subject motivation. A concern could be that subjects may have been, with time, simply less motivated in general to move or perform maximal explosive tasks. However, there was a marginal, non-significant, increase in our measure of subject motivation. *After* isolation, the up-tick in neuromuscular performance compared to during isolation could, however, be associated with the parallel up-tick in subject motivational state. Also, the discordant changes between upper and lower-limbs on neuromuscular testing, rather than similar changes in both, further suggests that subject motivation or factors such as repeated testing is likely not the mediating factor.

The current study had some important limitations. Firstly, we did not have a control group that performed testing on the same schedule but did not undergo isolation. Comparable data are also not available in the literature. Also, the number of subjects was limited for logistical reasons. Despite this we were able to find significant effects by conducting a number of measures during the course of confinement, though ability to generalise to a wider population would be limited. Subjects also performed an exercise program during confinement. The type of exercise and order of their performance fixed according to the study protocol, but subjects did not always comply with the requested volume of exercise and detailed information on exercise actually executed was not made available to researchers participating in the study. Nonetheless, neuromuscular or motivational state variables did not differ significantly between the phases when these exercise programs were due to be performed. Average gait speed and distance walked were significantly increased when subjects were scheduled to perform running exercises (*data not shown*). Importantly, we found significant decrements in neuromuscular performance *despite* attempts to implement a countermeasure exercise program.

In conclusion the current study investigated physical inactivity and neuromuscular performance in prolonged 520 d confinement. Overall, the data suggest that prolonged confinement is a form of reduced physical activity with concurrent adaptation in lower-limb, rather than upper-limb, neuromuscular performance.

## References

[1] Palinkas LA, Keeton KE, Shea C, Leveton LB (2011) Psychosocial Characteristics of Optimum Performance in Isolated and Confined Environments. Washington, DC, USA.

[2] Sauer J, Wastell DG, Hockey GR (1996) Skill maintenance in extended spaceflight: a human factors analysis of space and analogue work environments. Acta Astronaut 39: 579–587. 11540781 10.1016/s0094-5765(97)00006-4

[3] Adams OS, Chiles WD (1961) Human performance a function of the work-rest ratio during prolonged confinment. Marietta, Georgia, USA. 10.21236/ad0273511 24546909

[4] Shimamiya T, Terada N, Hiejima Y, Wakabayashi S, Kasai H, et al (2004) Effects of 10-day confinement on the immune system and psychological aspects in humans. J Appl Physiol 97: 920–924. 15145927 10.1152/japplphysiol.00043.2004

[5] Palinkas LA, Houseal M (2000) Stages of change in mood and behavior during a winter in Antarctica. Environ Behav 32: 128–141. 11542941 10.1177/00139160021972469

[6] Luria T, Matsliah Y, Adir Y, Josephy N, Moran DS, et al (2010) Effects of a prolonged submersion on bone strength and metabolism in young healthy submariners. Calcif Tissue Int 86: 8–13. 19882096 10.1007/s00223-009-9308-9

[7] Fox KM, Magaziner J, Hawkes WG, Yu-Yahiro J, Hebel JR, et al (2000) Loss of bone density and lean body mass after hip fracture. Osteoporos Int 11: 31–35. 10663356 10.1007/s001980050003

[8] Jørgensen L, Crabtree NJ, Reeve J, Jacobsen BK (2000) Ambulatory level and asymmetrical weight bearing after stroke affects bone loss in the upper and lower part of the femoral neck differently: bone adaptation after decreased mechanical loading. Bone 27: 701–707. 11062359 10.1016/s8756-3282(00)00374-4

[9] Giangregorio LM, Hicks AL, Webber CE, Phillips SM, Craven BC, et al (2005) Body weight supported treadmill training in acute spinal cord injury: impact on muscle and bone. Spinal Cord 43: 649–657. 15968302 10.1038/sj.sc.3101774

[10] Pavy-Le Traon A, Heer M, Narici MV, Rittweger J, Vernikos J (2007) From space to Earth: advances in human physiology from 20 years of bed rest studies (1986–2006). Eur J Appl Physiol 101: 143–194. 17661073 10.1007/s00421-007-0474-z

[11] Izquierdo M, Aguado X, Gonzalez R, López JL, Häkkinen K (1999) Maximal and explosive force production capacity and balance performance in men of different ages. Eur J Appl Physiol Occup Physiol 79: 260–267. 10048631 10.1007/s004210050504

[12] Schimpl M, Moore C, Lederer C, Neuhaus A, Sambrook J, et al (2011) Association between walking speed and age in healthy, free-living individuals using mobile accelerometry–a cross-sectional study. PLoS One 6: e23299. 21853107 10.1371/journal.pone.0023299 PMC3154324

[13] Schimpl M, Lederer C, Daumer M (2011) Development and validation of a new method to measure walking speed in free-living environments using the actibelt(R) platform. PLoS One 6: e23080. 21850254 10.1371/journal.pone.0023080 PMC3151278

[14] Farley CT, Morgenroth DC (1999) Leg stiffness primarily depends on ankle stiffness during human hopping. J Biomech 32: 267–273. 10093026 10.1016/s0021-9290(98)00170-5

[15] Incel NA, Ceceli E, Durukan PB, Erdem HR, Yorgancioglu ZR (2002) Grip Strength: Effect of Hand Dominance. Singapore Med J 43: 234–237. 12188074

[16] Schneider S, Askew CD, Brümmer V, Kleinert J, Guardiera S, et al (2009) The effect of parabolic flight on perceived physical, motivational and psychological state in men and women: correlation with neuroendocrine stress parameters and electrocortical activity. Stress 12: 336–349. 19006009 10.1080/10253890802499175

[17] Pinheiro JC, Bates DM (2000) Mixed-effects models in S and S-PLUS. Chambers J, Eddy W, Haerdle W, Sheather S, L. T, editors Berlin: Springer. 528 p p.

[18] Brunner E, Domhof S, Langer F (2002) Nonparametric Analysis of Longitudinal Data in Factorial Experiments. New York: Wiley. 288 p p.

[19] Faul F, Erdfelder E, Lang A-G, Buchner A (2007) G*Power 3: A flexible statistical power analysis for the social, behavioral, and biomedical sciences. Behav Res Methods 39: 175–191. 17695343 10.3758/bf03193146

[20] Le Blanc A, Schneider V, Shackelford L, West S, Oganov V, et al (2000) Bone mineral and lean tissue loss after long duration space flight. J Musculoskelet Neuronal Interact 1: 157–160. 15758512

[21] Michaelis I, Kwiet A, Gast U, Boshof A, Antvorskov T, et al (2008) Decline of specific peak jumping power with age in master runners. J Musculoskelet Neuronal Interact 8: 64–70. 18398267

[22] Runge M, Rittweger J, Russo CR, Schiessl H, Felsenberg D (2004) Is muscle power output a key factor in the age-related decline in physical performance? A comparison of muscle cross section, chair-rising test and jumping power. Clin Physiol Funct Imaging 24: 335–340. 15522042 10.1111/j.1475-097X.2004.00567.x

[23] Pallafacchina G, Blaauw B, Schiaffino S (2012) Role of satellite cells in muscle growth and maintenance of muscle mass. Nutr Metab Cardiovasc Dis. doi:10.1016/j.numecd.2012.02.002. 10.1016/j.numecd.2012.02.002 22621743

[24] Guo B-S, Cheung K-K, Yeung SS, Zhang B-T, Yeung EW (2012) Electrical stimulation influences satellite cell proliferation and apoptosis in unloading-induced muscle atrophy in mice. PLoS ONE 7: e30348 doi:10.1371/journal.pone.0030348. 22253929 10.1371/journal.pone.0030348 PMC3257250

[25] Powers SK, Kavazis AN, McClung JM (2007) Oxidative stress and disuse muscle atrophy. J Appl Physiol 102: 2389–2397 doi:10.1152/japplphysiol.01202.2006. 17289908 10.1152/japplphysiol.01202.2006

[26] Finkel T, Holbrook NJ (2000) Oxidants, oxidative stress and the biology of ageing. Nature 408: 239–247 doi:10.1038/35041687. 11089981 10.1038/35041687

